# Application of Body Mass Index According to Height-Age in Short and Tall Children

**DOI:** 10.1371/journal.pone.0072068

**Published:** 2013-08-07

**Authors:** Marjolein Bonthuis, Kitty J. Jager, Ameen Abu-Hanna, Enrico Verrina, Franz Schaefer, Karlijn J. van Stralen

**Affiliations:** 1 European Registry for Paediatric Nephrology/ERA-EDTA Registry, Department of Medical Informatics, Academic Medical Center, University of Amsterdam, Amsterdam, The Netherlands; 2 Department of Medical Informatics, Academic Medical Center, University of Amsterdam, Amsterdam, The Netherlands; 3 Department of Pediatric Nephrology, Gaslini Children’s Hospital, Genoa, Italy; 4 Department of Pediatric Nephrology, University Children’s Hospital, Heidelberg, Germany; University of Sao Paulo, Brazil

## Abstract

**Background:**

In children with either delayed or accelerated growth, expressing the body mass index (BMI) to chronological age might lead to invalid body composition estimates. Reference to height-age has been suggested for such populations; however its validity has not been demonstrated.

**Methods:**

Anthropometric data of healthy children were obtained from the German KiGGS survey. We selected three samples with different height distributions representing short stature (mean height SDS: -1.6), normal stature (height SDS: 0), and tall stature (height SDS: +1.6), and compared BMI-for-age and BMI-for-height-age between these samples across the paediatric age range. Differences between samples were tested using Kruskal-Wallis one-way analysis of variance and permutation tests.

**Results:**

At a given age, BMI was distributed towards lower values in short, and towards higher values in tall subjects as compared to a population with average height distribution. Expressing BMI to height-age eliminated these differences in boys with a short stature from 4 years to 14 years of age, in tall boys from 4 to 16 years, in short girls aged 2-10 years or tall girls aged 2-17 years.

**Conclusion:**

From late infancy to adolescent age, BMI distribution co-varies with height distribution and referencing to height-age appears appropriate within this age period. However, caution is needed when data about pubertal status are absent.

## Introduction

The global childhood obesity epidemic and the associated cardiovascular burden in later life increase the need for a valid measure of childhood adiposity [1]. Ideally, adiposity should be defined based on body fatness, but such measurements are usually unfeasible both in clinical practice and in epidemiological studies [2]. Instead, the body mass index (BMI) is a straightforward measure of the weight-for–height ratio that is applicable throughout human lifespan [3].

In children, BMI is dependent on age, sex, and sexual maturation, and is therefore expressed as standard deviation scores (SDS) using age- and sex specific reference values [4]. When linear growth and/or maturity are affected by chronic illness (e.g. chronic kidney disease (CKD), Turner syndrome, or Marfan syndrome) the relationship between age, height, and sexual maturation may be altered. This might lead to an underestimation (in short children) or overestimation (in tall children) of the BMI compared to peers of the same chronological age [5]. Therefore, it has been suggested to express the BMI according to height-age (i.e. the age at which a child’s given height would be at the 50^th^ percentile) in short children [6]. Furthermore, the BMI of healthy tall children was found to be higher than the BMI of shorter children, yielding systematically higher overweight prevalence estimates among taller children [7,8]. These findings question the validity of referencing BMI to chronological age without accounting for relative height.

A recent study suggested that in children with CKD from 5 years up to adolescent age the BMI-for-height-age reflected physical development in the same way as BMI-for-age did in healthy children [9]. Therefore, it was concluded that BMI-for-height-age is the preferred method in childhood CKD. Although BMI-for-height-age might be an appropriate indicator of nutritional status in such children, information on the validity of the height-age approach over the entire childhood period, including pubertal and post-pubertal phase, and on its generalizability to tall populations is lacking. Therefore, we studied a large sample of healthy children to explore the impact of relative body size on the distribution of BMI-for-age and BMI-for-height-age across the paediatric age range.

## Methods

### Subjects

Anthropometric data were obtained from the German Health Interview and Examination Survey for children and adolescents (KiGGS) [10]. The sample has been described in detail elsewhere [11]. In summary, KiGGS is a cross-sectional nationally representative survey of 17 641 healthy German children aged 0.25-17 years conducted between May 2003 and May 2006. KiGGS used a two-stage sampling strategy. First, a sample of 167 communities, representative for community type and size in Germany, was drawn, followed by a random selection of equally sized cohorts of children per birth year from local population registries [11]. The survey involved self-administered questionnaires, physical examinations and tests, as well as a computer assisted personal interview performed by a physician. Standardized height and weight measurements were performed by trained staff members. Children with medical conditions (e.g. premature birth (only 0-1 year olds), severe infections (only 0-1 year olds), and chronic diseases) or the intake of medication (e.g. growth hormone, corticosteroids) possibly affecting growth were excluded [12], therefore, short stature children will most likely have idiopathic short statures.

Due to the small number of available children younger than 1 year of age the current analysis was restricted to children aged 1-17 years, resulting in a sample of 16,564 children.

### Definition of variables

Height SDS was calculated as follows: SDS = (individual patient values – mean values for age and sex-matched healthy peers)/SD values for age and sex-matched healthy peers [13].

BMI was calculated as weight/height^2^ and referred to either chronological age (BMI-for-age) or height-age (BMI-for-height-age).

### Statistical analysis

From the original KiGGS Survey, we selected three random samples of children based on their height distributions. In order to reflect a population with retarded growth, the first random sample had a mean height SDS of -1.6. Furthermore, starting from the original sample, we selected a sample with a mean height SDS of zero (e.g. matching a healthy reference population) and a tall sample with a mean height SDS of +1.6. No selection was made for age or sex, and each child was principally eligible to be selected in each sample. As this might have resulted in possible overlap between the samples, we performed sensitivity analyses including unique subjects in each sample.

We modelled age- and sex-specific median BMI curves for the three samples using the L (lambda; skewness in the data), M (median BMI), and S (sigma; coefficient of variation) method [4] and smoothed the curves using a 3-degree polynomial spline function with zero knots. The LMS method provides a method to normalize skewed anthropometric data by using a power transformation to remove the skewness. To take into account the relative precision of the estimates at each age, the samples were weighted according to the number of subjects and the BMI variation within each age group [4].

To examine intergroup differences in BMI distributions we used Kruskal-Wallis one-way analysis of variance. For every year of age we calculated the difference in the Kruskal-Wallis statistic for BMI-for-age and for BMI-for-height-age. The method that results in the smaller sum of Kruskal-Wallis statistics over the ages is the preferred method. To test whether the sum of Kruskal-Wallis statistics in the two groups significantly differed, we performed a permutation test (with 1000 permutations), in which the two Kruskal-Wallis statistics per age were randomly assigned to the BMI-for-age or the BMI-for-height-age group. Per permutation the sum of differences of the Kruskall-Wallis statistics over the ages was calculated. The distribution of these differences over all permutations was used to determine the *P*-value. *P*-values < 0.05 were considered statistically significant.

Similar to methods for blood pressure standardization in children as reported by the NHBPEP [14], we aimed to develop a formula for the LMS parameters needed to construct BMI reference curves, adjusting BMI for stature.

All statistical analyses were performed using SAS version 9.2 (SAS Institute Inc., Cary, NC, USA).

## Results

### Subjects

Characteristics of the subjects in the three samples are displayed in Table 1. All samples included a slightly lower number of boys than girls (41-45%) (NS). Mean age was 10.6 ± 4.7 years in the short sample, 9.6 ± 4.7 years in the sample with average height, and 7.9 ± 5.0 years in the tall sample (*P*<0.0001). The mean height-age was 8.1 ± 4.0 years, 9.0 ± 4.6 years, and 8.7 ± 5.6 years in the short, normal, and tall sample, respectively (*P*=0.0006).

**Table 1:** Subject characteristics by height distribution.

	**-1.6 height SDS** (N=755)	**0 height SDS** (N=1493)	**+ 1.6 height SDS** (N=756)
% male	45.2	43.0	42.3
Mean age (SD)*	10.6 (4.7)	9.6 (4.7)	7.9 (5.0)
Mean height-age (SD)*	8.1 (4.0)	9.0 (4.6)	8.7 (5.6)
Mean height SDS (SD)	-1.62 (0.80)	-0.01 (1.00)	1.61 (1.00)

* Significant difference on the 0.05 level

### BMI distribution by height SDS

**Figure 1:**
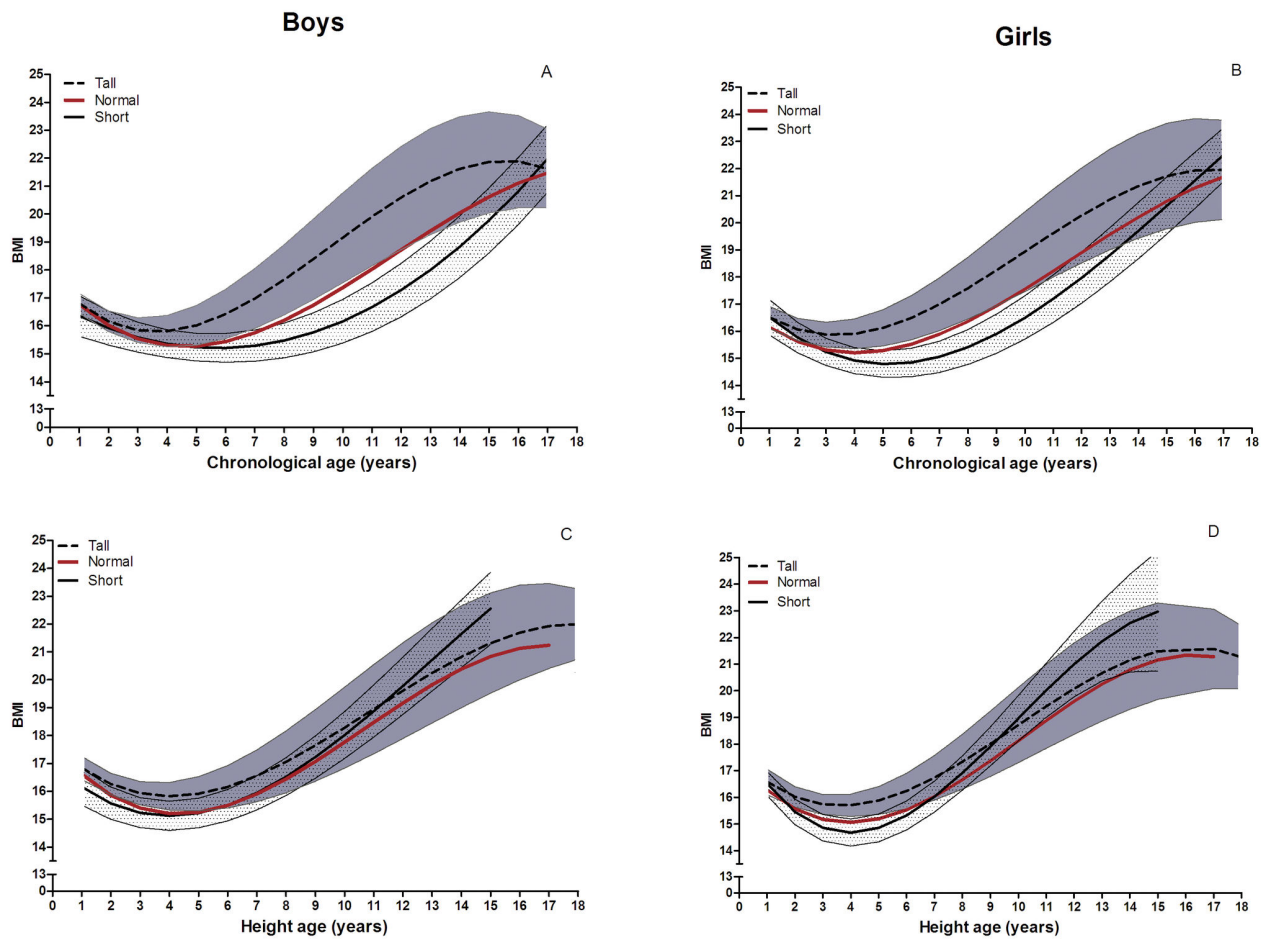
Smoothed median BMI curves for the short (mean height SDS -1.6), normal (mean height SDS 0), and tall (mean height SDS +1.6) sample. BMI according to chronological age for boys (A), girls (B) and BMI according to height-age for boys (C) and girls (D). The dotted lines and the shaded area represent the median BMI and confidence intervals of the tall sample and the short sample, respectively.

As shown in Figure 1A (boys) and 1B (girls) the median BMI (relative to chronological age) differed according to height SDS. In the infancy period the median BMI-for-age was similar irrespective of the child’s relative body size, whereas with increasing age BMI curves diverged until adolescent age, with smaller children showing consistently lower and taller children showing higher median BMI values within this age period. During adolescent age the three BMI curves converged. Expression of BMI relative to height-age markedly attenuated the variation of median BMI across the three samples both in boys (Figure 1C) and in girls (Figure 1D).

Based on the Kruskal-Wallis statistics, the differences in BMI distributions across the samples were smaller for BMI-for-height-age, and hence better, compared to BMI-for-age. The sum of differences in Kruskal-Wallis statistics was 62.85 for boys (Table 2) and 43.86 for girls (Table 3). For both sexes, the differences between the samples were significantly larger for BMI-for-age as compared to BMI-for-height-age (*P*=0.019 for boys and *P*=0.036 for girls).

**Table 2:** Differences in Kruskal-Wallis statistics to identify intergroup differences between the samples for BMI according to chronological age and BMI according to height-age in boys.

**Age (years)**	**Kruskal-Wallis statistic BMI-for-chronological age**	**Kruskal-Wallis statistic BMI-for-height-age**	**Difference between Kruskal-Wallis statistics**
1	3.77	4.99	-1.22
2	1.79	1.97	-0.18
3	8.71	13.04	-4.33
4	1.04	8.04	-7.00
5	11.08	3.33	7.75
6	8.54	3.92	4.62
7	5.31	2.10	3.21
8	16.21	2.03	14.18
9	10.38	3.91	6.47
10	7.63	0.89	6.74
11	9.51	0.37	9.14
12	1.79	4.66	-2.87
13	22.63	1.06	21.57
14	2.11	1.63	0.48
15	0.07	1.69	-1.62
16	5.90	0.20	5.70
17	2.94	2.73	0.21
Total			62.85*

* *P*=0.019

To test whether the median BMI differed according to height-SDS we calculated the Kruskal-Wallis statistics (KW). In order to examine whether BMI-for-age would result in more similar BMI distributions than BMI-for-height-age, per age group differences between the KWs were calculated and summed. Subsequently, a permutation test was used to test whether these differences were statistically significant. A positive difference in KW means that height related BMI differences were larger for BMI-for-age as compared to BMI-for-height-age.

**Table 3:** Differences in Kruskal-Wallis statistics to identify intergroup differences between the samples for BMI according to chronological age and BMI according to height-age in girls.

**Age (years)**	**Kruskal-Wallis statistic BMI-for-chronological age**	**Kruskal-Wallis statistic BMI-for-height-age**	**Difference between Kruskal-Wallis statistics**
1	1.15	1.29	-0.14
2	17.77	3.84	13.93
3	3.08	15.78	-12.70
4	18.92	10.73	8.19
5	9.04	6.32	2.72
6	4.11	0.37	3.74
7	3.92	2.24	1.68
8	12.12	0.41	11.71
9	9.72	4.21	5.51
10	13.81	2.80	11.01
11	15.30	13.04	2.26
12	1.63	0.97	0.66
13	6.82	1.73	5.09
14	1.51	5.62	-4.11
15	2.27	1.10	1.17
16	0.31	0.77	-0.46
17	4.59	0.99	3.60
Summed			43.86*

* *P*=0.036

To test whether the median BMI differed according to height-SDS we calculated the Kruskal-Wallis statistics (KW). In order to examine whether BMI-for-age would result in more similar BMI distributions than BMI-for-height-age, per age group differences between the KWs were calculated and summed. Subsequently a permutation test was used to test whether these differences were statistically significant. A positive difference in KW means that height related BMI differences were larger for BMI-for-age as compared to BMI-for-height-age.

**Figure 2:**
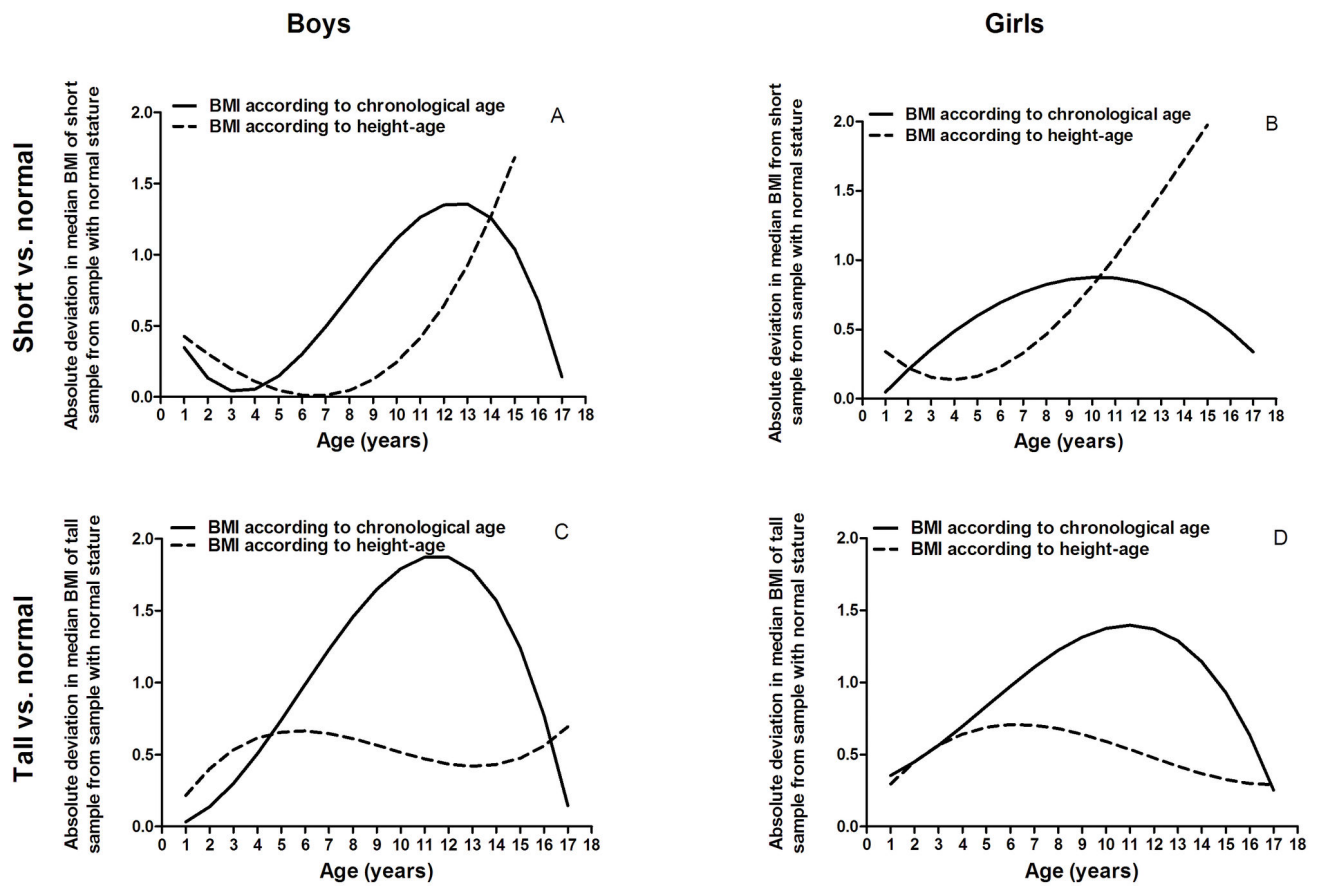
Absolute deviation in median BMI-for-chronological-age and BMI-for-height-age between samples with tall or short stature from the sample with normal stature. A smaller deviation indicates that the median BMI of short or tall sample is more similar to the median BMI of the sample with normal stature. For boys with a short stature in the ages 4 up to 14 years of age, expressing BMI according to height-age resulted in a smaller deviation from the BMI distribution of children with a normal stature than when expressing BMI to chronological age, whereas in boys with a tall stature this was true in the ages 4-16 years. In girls, expressing BMI to height-age resulted in a smaller deviation in the ages 2-10 years if of being short stature and in the ages 2-17 years when being tall.

In order to visualize which method yielded more similar median BMI curves across the samples, we calculated the (absolute) deviation in median BMI of the samples with tall and short statures from the sample with average height distribution for BMI-for-age and BMI-for-height-age (Figure 2). A smaller absolute deviation for BMI-for-height-age than the absolute deviation for BMI-for-age indicates that the median BMI of short and tall samples were more similar to the median BMI of the sample with normal stature when using BMI-for-height-age. The crossing points of the curves indicate the age boundaries within which BMI-for-height-age in short and tall children yielded BMI distributions more similar to that of children with average height than BMI-for-age (Figure 2). In boys with a short stature, differences were marginal up to the age of 4 years, but from 4 to 14 years BMI-for-height-age showed smaller deviations from normal stature population than BMI-for-chronological age. Beyond age 15 years BMI-for-height-age was not applicable. In boys with a tall stature BMI-for-height-age resulted in smaller deviation from the sample with average height from age 4-16 years, with small differences before age 4 years. In short girls, BMI-for-height-age was superior until age 10 years and in tall girls until age 17 years.

The sensitivity analyses using non-overlapping samples did not reveal different results.

### Formula based on LMS parameters for construction of BMI reference curves

Besides differences in median (M) BMI across the samples, we found relatively similar values for the S parameters (average absolute difference between three samples 0.02 for both sexes) and a strong variation in the L parameter (absolute average variation: 0.86 for boys and 0.80 for girls). These findings suggest that BMI distributions of the three samples mainly differ in their median and their level of skewness. As an alternative method we tried to develop a single formula to estimate L accounting for relative stature. However, we were unable to establish a clear trend for L and hence adjustment for the skewness in BMI according to body height was not possible.

## Discussion

BMI-for-age clearly differed according to stature. Compared to subjects with a normal stature, BMI was systematically higher in tall and lower in short subjects. In infants and adolescents height SDS related BMI differences were marginal, whereas between these periods large BMI differences were found. The use of BMI-for-height-age largely abolishes these systematic differences.

Both body size and pubertal status are important to consider when assessing body composition, as well as other physiological measures, in children [9,15–17]. The observed positive relationship between BMI and height SDS during pre-pubertal age is compatible with an effect of the maturational tempo on body composition [18]. Given the clear effects in children at the ends of the normal height spectrum and the positive association between height and BMI observed in healthy children [7,8], it can be assumed that the differences would be even stronger in subjects with more abnormal statures as observed in disease states.

While the use of body height rather than age as a reference corrects for the effect of relative body size throughout most of the pre-pubertal period [19], it is important to consider that the height-age approach is invalid when delayed or accelerated growth and pubertal development are discordant, such as in precocious puberty, and from the time that final height is achieved [9].

By definition, short subjects will have lower height-ages than chronological ages, resulting in a rather limited number of short children with height ages above 14 years in this study. Although we corrected the BMI curves for the number of subjects per age group, this resulted in wider confidence intervals according to height-age in short subjects of adolescent age. Furthermore, the greater physiological variation in BMI during adolescence results in larger confidence intervals during this period. These factors limited our ability to define a precise age up to which the use of BMI-for-height-age will result in a more valid expression of adiposity than the BMI-for-chronological-age. In early infancy, the rapid change and high variability of linear growth and relative obesity [12,20] appear to preclude any advantage of referencing to height-age; in this period, differences were generally marginal. Within the limits of sensitivity given by our approach, the use of BMI-for-height-age appears preferable in short boys from 4 to 14 years, in tall boys from 4 to 16 years, in short girls from 2 to 10 years, and in tall girls from 2–17 years, because within these age periods height-related differences between BMI-for-height-age were substantially smaller than differences between BMI-for-chronological age. Since the differences in the infantile age period were negligible, we propose to reference BMI to height-age in short girls and boys up to the age of 10 and 14 years, respectively, and in tall children up to the age of 17 years.

In summary, BMI-for-age differed according to height SDS, with taller children showing systematically higher and shorter children showing lower median BMI values. As a rule of thumb, when using BMI in children with abnormal statures expressing BMI to height-age should be the preferred method in the ages up to 10 and 14 years in short girls and boys and up to the age of 17 years in tall children. Beyond these age limits BMI-for-age is likely to result in a more valid estimation, but caution is needed in the absence of data on pubertal stage and achievement of final height.
